# A biomechanical study of plate versus intramedullary devices for midshaft clavicle fixation

**DOI:** 10.1186/1749-799X-3-28

**Published:** 2008-07-16

**Authors:** S Raymond Golish, Jason A Oliviero, Eric I Francke, Mark D Miller

**Affiliations:** 1 Department of Orthopaedic Surgery, University of Virginia Health System, Charlottesville, Virginia, USA

## Abstract

**Background:**

Non-operative management of midshaft clavicle fractures is standard; however, surgical management is increasing. The purpose of this study is to compare the biomechanical performance of plate versus intramedullary fixation in cyclic bending for matched pairs of cadaveric clavicles. We hypothesized that the biomechanical properties are similar.

**Methods:**

Eight sets of matched clavicles with vertical, midshaft osteotomies were prepared from fresh, frozen cadavers. A 3.5 mm dynamic compression plate or a 3.8 or 4.5 mm intramedullary device were used for fixation. Clavicles were loaded in a four-point bend at 6 different loads for 3000 cycles at 1 Hz starting with 180 N and increasing by 180 N with sampling at 2 Hz. Failure was defined as 10 mm of displacement or catastrophic construct failure prior to 10 mm of displacement.

**Results:**

Between constructs, there was a significant difference with large effect size in displacement at fixed loads of 180 N (*P *= 0.001; Cohen's d = 1.85), 360 N (*P *= 0.033; Cohen's d = 1.39), 540 N (*P *= 0.003; Cohen's d = 0.73) and 720 N (*P *= 0.018; Cohen's d = 0.72). There was a significant difference with large effect size in load at fixed displacements of 5 mm (*P *= 0.001; Cohen's d = 1.49), 7.5 mm (*P *= 0.011; Cohen's d = 1.06), and 10 mm (*P *= 0.026; Cohen's d = 0.84).

**Conclusion:**

Plate constructs are superior in showing less displacement at fixed loads and greater loads at fixed displacements over a broad range of loads and displacements with cyclic four-point bending. The clinical relevance is that plate fixation may provide a stronger construct for early rehabilitation protocols that focus on repetitive movements in the early pre-operative period.

## Background

Clavicle fractures comprise 5–10% of all skeletal fractures, and 80% of these occur in the middle-third [1]. These fractures result from a blow to the shoulder causing axial loading [2]. The standard of care is closed treatment with sling and swathe; however, recent studies have found higher rates of delayed union, nonunion, shoulder pain, and shoulder weakness with non-operative care [3]. Risk factors for poor outcome with non-operative treatment include shortening, initial displacement, fracture comminution, and age. The indications for surgery include the need for earlier functional mobilization in the patient with an isolated injury [4] in addition to open fractures and the polytraumatized patient.

For operative treatment, open reduction and internal fixation with a 3.5 mm dynamic compression plate is the standard; however, intramedullary fixation is a less invasive alternative. A retrospective clinical study by Wu et al. compared plate to IM fixation for aseptic nonunions [5]. A biomechanical study by Proubasta et al. compared a 6-hole 3.5 mm dynamic compression plate to a 4.5 mm intramedullary Herbert screw [6].

Clinically, cyclic bending is a likely model of construct failure, especially in the setting of early functional mobilization. Loosening at the bone-implant interface, fatigue failure, and broken hardware are clinical anecdotes shared by surgeons caring for patients with these injuries. No biomechanical study has compared plate to intramedullary fixation in cyclic bending. The purpose of this study is to compare plate fixation versus intramedullary fixation in cyclically loaded clavicles in a four-point bend with sequentially increasing loads. We hypothesized that the biomechanical properties are similar.

## Methods

Eight sets of paired clavicles were obtained from 8 fresh, frozen cadavers. There were 5 male and 3 female specimens in the age range 37 to 68 years. Vertical osteotomies were made at the midpoint using an oscillating saw. A single clavicle from each matched pair set was randomly assigned to either the intramedullary fixation technique or the plating technique, and the contralateral clavicle was assigned to the other fixation technique.

Seven-hole, 3.5 mm titanium dynamic compression plates (ACE/Depuy, Warsaw, IN, USA) were contoured to the superior surface of reduced clavicles. The plate size was chosen to be the largest width that would contour smoothly to all cadavers samples without hardware prominence beyond the cortical borders. AO technique was used to place 3.5 mm cortical, fully threaded titanium screws; holes were drilled, tapped, and measured prior to screw selection. Six cortices of fixation were acquired on either side of the osteotomy, after positioning the middle hole of the plate over the osteotomy site to balance three holes evenly on either side. The Rockwood Pin (Depuy, Warsaw, IN, USA) was used as the intramedullary device to fix the osteomized clavicles. Using the technique recommended by the implant manufacturer, clavicles were repaired using either a 3.8 mm pin or a 4.5 mm pin depending on bone diameter. An attempt to insert a 4.5 mm pin was made, but if the pin proved too large to pass without binding or fracture, a 3.8 mm pin was passed. Five pins of 4.5 mm width and three pins of 3.8 mm width were used.

**Figure 1:**
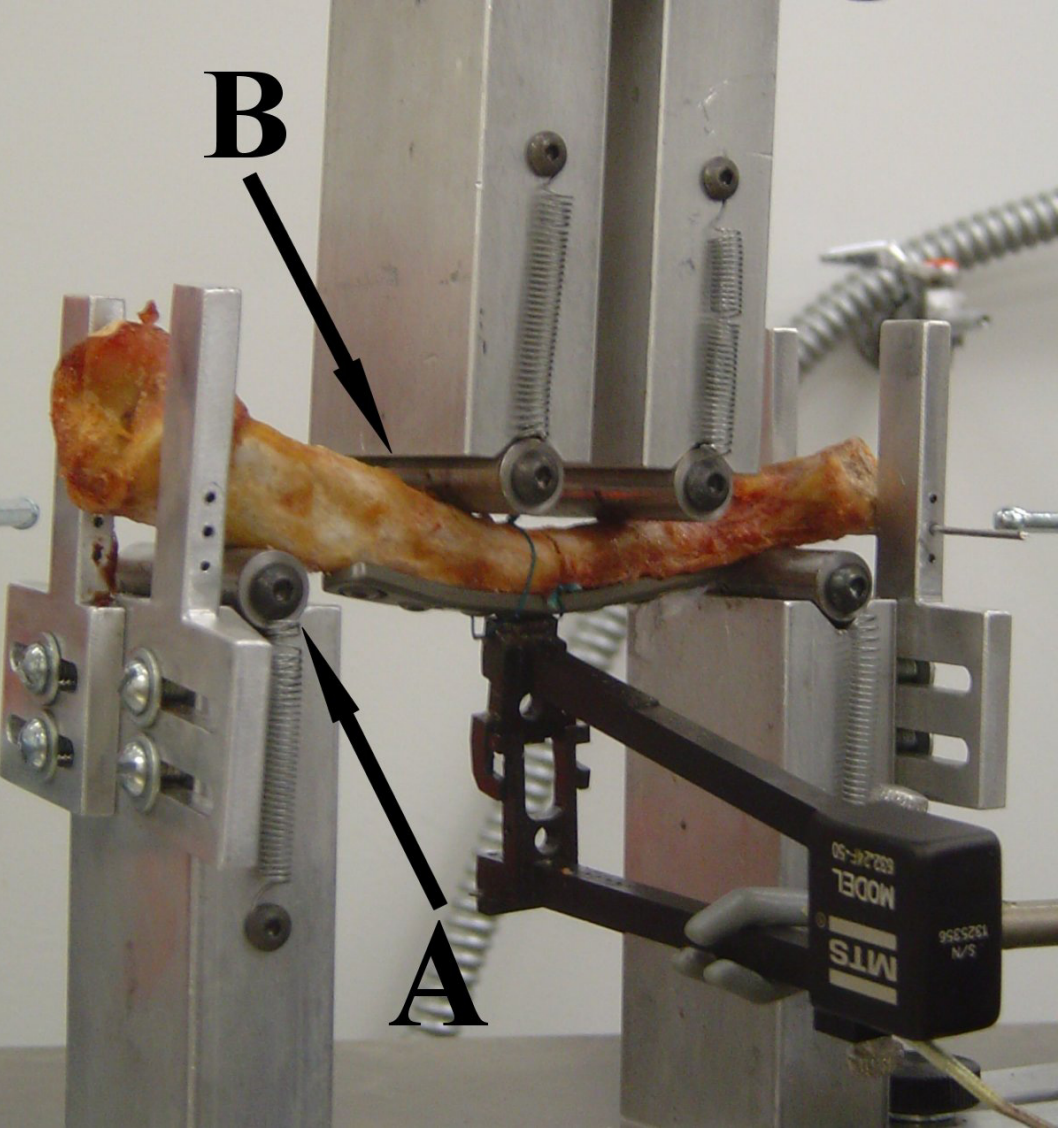
**Photograph of clavicle loaded in jig for testing by cyclic loading in a four-point bend.** Label A denotes the outer arm. Label B denotes the inner arm.

A custom jig consisting of 2 superior inner arms and 2 inferior outer arms was constructed to load clavicles in a four-point bend. Figure 1 is a photograph of the jig loaded with a clavicle for testing. The clavicles and arms were optimally positioned such that the osteotomy site was at the midpoint between both the inner and outer arms. Clavicles were placed with the superior surface contacting the outer arms and the inferior surface contacting the inner arms, loading the superior surface of the clavicle in tension. In order to stabilize this position during loading, two 0.157 mm K-wires were placed thru the jig and the clavicle's acromial end.

The MTS-Bionix (Materials Testing Systems, Minneapolis, MN, USA) servohydraulic testing machine with a 454 kg load cell was used to load the clavicles while a digital extensometer measured displacement. The clavicles were loaded for 3000 cycles at a frequency of 1 Hz starting with a force of 180N. The loading cycles of 3000 repeats were incrementally increased by 180N such that the clavicles were tested at 180N, 360N, 540N, 720N, 900N, 1080N loads. The extensometer measured the peak and valley of each load cycle with a sampling rate of 2 Hz. Testing was stopped after displacement of greater than or equal to 10 mm occurred, after catastrophic failure of the construct at the bone-implant interface, or at the completion of the 1080N loading cycle. Data analysis was performed with SPSS 12.0 (SPSS Inc., Chicago, IL, USA) using paired t-tests, Wilcoxon signed-rank tests, chi-squared tests, and Bonferroni-Sidak multitest correction. An alpha value (type I error rate) of 0.05 was considered significant. Power analysis was performed with a beta value (type II error rate) of 0.20 in order to choose the sample size of 8 matched clavicle pairs as detailed in the results section.

## Results

Eight matched pairs of clavicles ranged in length from 147 mm to 175 mm and ranged in diameter from 11 mm to 16 mm. The diameter was measured at the widest point in the middle one-third of the bone. Table 1 summarizes the clavicle and test characteristics. Between matched pairs of clavicles, there was no significant difference in clavicle length (*P *= 0.99), diameter (*P *= 0.35), inner arm placement (*P *= 0.70) or outer arm placement (*P *= 0.41) by paired t-tests. Five right-sided clavicles received the plate-construct and 3 right-sided clavicles received the pin-construct; there was no significant difference in clavicle-laterality versus construct-type (*P *= 0.48) by chi-squared test.

**Table 1:** Summary of clavicle and test characteristics.

Clavicle Pair	Plate Size (mm)	Pin Size (cm)	Plate/Pin Side	Length (mm)	Diameter (mm)	Inner Point (mm)	Outer Point (mm)
1	3.5	3.8	R/L	147.5	13.0	35.5	105.0
2	3.5	4.5	L/R	153.0	16.0	36.0	109.5
3	3.5	3.8	R/L	149.5	11.5	35.5	106.5
4	3.5	3.8	R/L	155.0	13.0	40.0	112.0
5	3.5	4.5	R/L	175.0	15.0	37.0	123.0
6	3.5	4.5	L/R	164.0	16.0	37.0	116.0
7	3.5	4.5	R/L	152.0	14.0	37.0	113.0
8	3.5	4.5	L/R	165.0	16.0	37.0	113.0

Clavicle length, diameter, inner arm position, and outer arm position are averages of two matched clavicles.

Loads were measured for serial displacements for both constructs. Table 2 summarizes the data. For all displacements, the plate constructs had lower mean load. There was a significant difference between plate versus pin constructs for displacements of 5 mm (*P *= 0.001; Cohen's d = 1.49)), 7.5 mm (*P *= 0.011; Cohen's d = 1.06), and 10 mm (*P *= 0.026; Cohen's d = 0.84) by paired t-tests with Bonferroni multi-test correction (corrected alpha = 0.029). The use of Sidak multitest correction or Wilcoxon signed-rank tests did not change the significance at any displacement.

**Table 2:** Loads for plate versus pin constructs at displacements of 5 mm, 7.5 mm and 10 mm.

Clavicle Pair	Load at 5 mm for plate (N)	Load at 5 mm for pin (N)	Load at 7.5 mm for plate (N)	Load at 7.5 mm for pin (N)	Load at 10 mm for plate (N)	Load at 10 mm for pin (N)
1	720	540	1080	900	1080	1080
2	540	360	720	360	720	360
3	720	540	720	720	720	720
4	720	360	720	360	720	540
5	720	540	900	720	900	900
6	720	540	720	720	720	720
7	540	360	540	360	540	360
8	900	360	> 1080	720	> 1080	900

Values are in Newtons (N). Differences are significant at 5 mm (p = 0.001), 7.5 mm (p = 0.011), and 10 mm (p = 0.026) by paired t-tests.

Displacements were measured for several loads for both constructs. Table 3 summarizes the data. For all loads, the plate constructs had lower mean displacement. There was a significant difference between plate versus pin constructs for loads of 180N (*P *= 0.001; Cohen's d = 1.85), 360N (*P *= 0.033; Cohen's d = 1.39), 540N (*P *= 0.003; Cohen's d = 0.73) and 720N (*P *= 0.018; Cohen's d = 0.72) by paired t-tests with Bonferroni multi-test correction (corrected alpha = 0.027). At loads of higher than 540N, half or more of all constructs had failed. There was no significant difference at 900N (*P *= 0.44 for N = 2 pairs), and there were no testable data pairs at 1080N. The use of Sidak multi-test correction or Wilcoxon signed-rank tests did not change the significance at any load.

**Table 3:** Displacements for plate versus pin constructs at various loads after all 3000 cycles have elapsed for the load.

Clavicle Pair	Displacement at 180N for plate	Displacement at 180N for pin	Displacement at 360N for plate	Displacement forat 360N pin	Displacement at 540N for plate	Displacement at 540N for pin	Displacement at 720N for plate	Displacement at 720N for pin
1	1.18	1.57	1.89	2.35	3.00	5.30	5.58	6.98
2	1.58	2.14	3.73	--	6.32	--	8.73	--
3	1.06	2.15	1.84	3.53	3.25	4.94	--	--
4	1.70	2.80	2.18	8.97	3.80	--	--	--
5	0.93	1.38	1.95	3.54	3.66	5.23	5.76	7.86
6	0.84	2.38	1.73	4.75	3.34	6.90	10.7	12.7
7	1.20	1.65	2.52	--	9.40	--	--	--
8	1.72	2.50	2.88	5.14	3.60	6.48	4.35	8.04

Values are in millimeters. Differences are significant at 180N (p = 0.001), 360N (p = 0.033), 540N (p = 0.003) and 720N (p = 0.018) by paired t-tests. At loads of higher than 720N, over half of constructs had failed.

The experiment was designed using statistical power analysis to choose eight cadaver pairs. The power of paired t-tests is 0.81 for N = 8 by Monte Carlo simulation of 2000 pseudorandom variates from a joint normal distribution with a large effect size (Cohen's d = 0.8).

## Discussion

The main finding of the present study is that plate constructs show less displacement at fixed loads and greater loads at fixed displacements over a broad range of loads and displacements with cyclic four-point bending. The clinical relevance is that plate fixation may provide a stronger construct for early rehabilitation protocols that focus on repetitive movements in the early pre-operative period.

During a midshaft clavicle fracture, the anterosuperior surface experiences tensile forces whereas the posteroinferior surface experiences compressive forces [7]. Proubasta et al. compared a 6-hole 3.5 mm dynamic compression plate to a 4.5 mm intramedullary Herbert screw [6]. They concluded that there was no difference in strength when constructs were non-cyclically loaded in a three-point bend with the load sequentially increased to failure. Harnroongroj et al. argued that superior plating is stronger than anterior plating for transverse clavicular fractures without a cortical defect [7]. Iannotti et al. tested osteotomized and plated clavicles in compression and torsion using superior versus anterior plating with 3.5 mm reconstruction plates, 3.5 mm limited contact dynamic compression plates, and 2.7 mm dynamic compression plates [8]. They concluded that superior plating with 3.5 mm limited contact dynamic compression plates provided the best stiffness, rigidity, and strength.

The present study focused on loosening at the bone-implant interface during cyclic bending as a putative mode of failure. Mechanical properties in static compression may affect re-injury due to an axial loading similar to a primary injury pattern. However, cyclic loading in a bending mode likely contributes to construct failure in the setting of early functional mobilization. Loosening at the bone-implant interface, fatigue failure, and broken hardware are clinical anecdotes shared by surgeons caring for patients with these injuries. In addition, repetitive bending versus repetitive torsion is more likely to be experienced in the immediate post-operative period (1 to 2 months) with a typical shoulder rehabilitation protocol focused on repetitive movements that avoid the extremes of motion, since torsional forces are increased at extreme forward flexion for example. The present study used load versus displacement as measures of construct integrity. In this testing context, stiffness need not be calculated from the stress strain curve, since neither linear elastic nor linear viscoelastic behavior is expected. Instead, a pattern of progressive construct failure is expected. In the present study, simple comparisons of displacement versus load and load versus displacement are reasonable surrogate measurements for a complex behavior.

There is limited clinical research directly comparing plating to intramedullary fixation for acute fractures of the clavicle. However, a study by Wu et al. retrospectively examined plating vs. intramedullary nailing for the treatment of clavicular nonunion [5]. The study showed an 18.2% nonunion rate with plating compared with 11.1% for nailing, the difference being attributed to the nail's resistance to compressive stresses. The authors concluded that plating provides better rotational stability, but this was not critical if the post-operative protocol limits forward flexion to 90 degrees for 1 to 2 months post-operatively. Several other studies have found intramedullary pinning to be at least equally effective as plating, especially for the treatment of nonunion, though without direct comparison [9,10].

Midshaft clavicle fractures are common, and conservative management results in approximately 5% nonunion rate [11]. While non-operative management remains the mainstay of treatment for most midshaft clavicle fractures, the indications for surgery may be expanding. A recent multicenter randomized controlled trial comparing plate fixation to conservative management demonstrated improved functional outcome and lower rates of nonunion and malunion at one year [12].

## Conclusion

For the fixation of midshaft clavicle fractures, plate constructs show less displacement at fixed loads and greater load at fixed displacements over a broad range of loads and displacements with cyclic four-point bending.

## Competing interests

The authors declare that they have no competing interests. No author has contractual obligations, consultant agreements, is a stockholder, or has other competing interests regarding ACE/Depuy, Warsaw, IN, USA which provided support.

## Authors' contributions

SRG contributed to analysis and interpretation of data, and to drafting the manuscript. JAO contributed to conception and design, to acquisition of data, and to drafting the manuscript. EIF contributed to conception and design, and to drafting the manuscript. MDM contributed to conception and design, to analysis and interpretation of data, and to revising the manuscript critically for important intellectual content. All authors have given final approval of the version to be published.
